# Whole maize flour could enhance food and nutrition security in Malawi

**DOI:** 10.1007/s44187-025-00311-y

**Published:** 2025-02-17

**Authors:** Theresa Nakoma Ngoma, Victor Taleon, Brighton M. Mvumi, Aggrey P. Gama, Natalia Palacios-Rojas, Limbikani Matumba

**Affiliations:** 1 Faculty of Life Sciences and Natural Resources, Lilongwe University of Agriculture and Natural Resources (LUANAR), Natural Resources College, Box 143, Lilongwe, Malawi; 2 HarvestPlus, Innovation Policy and Scaling Unit, International Food Policy Research Institute (IFPRI), 1201 Eye Street, Washington, NW, DC 20005 USA; 3 Department of Agricultural and Biosystems Engineering, Faculty of Agriculture Environment and Food Systems, University of Zimbabwe, Mt Pleasant, P. O. Box MP167, Harare, Zimbabwe; 4 Faculty of Food and Human Sciences, LUANAR, Bunda College, Box 219, Lilongwe, Malawi; 5 International Maize and Wheat Improvement Center (CIMMYT), Km. 45 Carretera Mexico-Veracruz, El Batan, 56130, 00174 Texcoco, Mexico

**Keywords:** Dehulling process, Food loss, Milling practices, Nutrient losses, Maize consumption, Maize flour

## Abstract

Maize is the staple cereal in Malawi, with a daily per capita consumption of 383 g (dry matter basis), primarily consumed in the form of *nsima*, a thick porridge. We combined a milling experiment with focus group discussions (FGDs) to provide insights into mass and nutrient losses during maize grain dehulling and maize flour consumption patterns in rural Malawi. Milling batches (30 kg) of four maize grain varieties were dehulled at three abrasive disk dehullers under controlled conditions. The impact of maize variety and dehuller design on mass and nutrient losses during dehulling was statistically significant (p < 0.05), with a mean mass loss of 28.1 ± 5.7%, and nutrient losses of 9.8 ± 1.9% for protein, 61.7 ± 2.0% for zinc, and 47.7 ± 3.6% for iron. Six FGDs conducted in rural areas of Lilongwe District revealed a preference for refined flour due to convenience and cultural norms, despite the nutritional benefits of whole grain flour, which was recognized for its ability to provide satiety, particularly during periods of maize scarcity. Participants also highlighted switching between flour types based on seasonal maize availability, social stigma associated with whole grain flour, and awareness of nutrient losses during dehulling. Given Malawi’s precarious food insecurity situation, transitioning from dehulled maize flour *nsima* to whole maize flour or less refined *nsima*, is imperative. Our study findings can have food and nutritional savings for other southern Africa countries where the dehulling is a common practice.

**Supplementary Information:**

The online version contains supplementary material available at 10.1007/s44187-025-00311-y.

## Introduction

Food insecurity continues to pose significant challenges in Malawi, with the country ranking 91th out of 113 nations on the Global Food Security Index [1]. Typically, households have maize, the country’s basic staple, lasting 6–7 months from each crop production season [2]. Despite the food shortage, most households consume maize after undergoing a dehulling process that may result in significant mass and nutrient losses [3]. This practice has the potential to exacerbate malnutrition.

Dehulling involves separating the pericarp and germ from the endosperm, often achieved through machinery like abrasive disk dehullers or traditional methods such as using a mortar and pestle [4]. The extent to which the pericarp and germ are separated varies significantly. Some methods remove the aleurone layer, which is rich in proteins, minerals, phytochemicals and antioxidants, along with the pericarp, while others retain part of it attached to the endosperm. This variability may be influenced by factors such as grain moisture, maize variety and type of dehuller used ultimately impacting the nutrient density of the refined flour produced [5–8]. Consequently, there are notable differences in mass loss and yield.

It has been estimated that 57% of households in Malawi produce their own maize flour [3]. In contrast, a recent study conducted in Lilongwe, Malawi’s capital, found that 89% of households utilize local abrasive disk dehullers and grinding mills for maize processing (Ngoma et. al., unpublished data). However, the effectiveness of these methods and their impact on mass and nutrient retention are uncertain. Maize milling generally leads to micronutrient losses [9, 10], with significant reductions in zinc concentration reported in maize meal from Malawi and Uganda [3, 9]. Although dehulling may not affect provitamin A carotenoids, it can decrease zinc and amino acid levels to different extents depending on the variety [5, 11].

The current is study investigated mass and nutrient losses during maize grain dehulling across different maize varieties and dehuller designs in Malawi, focusing on proteins, iron, and zinc due to their widespread deficiencies in the region [12, 13]. We also explored household practices, preferences, and challenges related to maize flour consumption. We hypothesized that: (1) maize variety and dehuller design, respectively, significantly affect both mass and nutrient losses, particularly for protein, iron, and zinc; and (2) maize flour consumption patterns in rural Malawi are influenced by seasonal availability, cultural norms, and trade-offs between nutritional value and convenience. The study findings have the potential to guide policy development and targeted interventions aimed at mitigating mass and nutrient losses, thus contributing to enhanced food and nutrition security in Malawi and other Southern African countries where dehulling is a common practice.

## Materials and methods

### Experimental analysis of mass and nutrient losses during maize dehulling

To evaluate the mass and nutrient losses during maize dehulling due to maize variety, an experiment with a replicated randomized complete block design was conducted. The experiment involved dehulling triplicate 30 kg batches of four distinct maize varieties commonly grown in Malawi. These included two flint varieties: an unspecified local variety procured from a farmer in Lilongwe District and *Pro Vit A (MH43A).* The other two were semi-flint varieties: *Kanyani* (SC403) and *Njobvu* (SC719). Hardness classifications were based on variety descriptions rather than direct measurements. All these four varieties were dehulled using three randomly selected abrasive disk dehullers in Lilongwe district. The dehullers were unbranded, locally fabricated, and featured distinct architectural designs, with variations in hoppers, dehulling chambers, and cylinders (Supplementary Fig. 1). To temper the maize, the 30 kg of maize was placed into a perforated tin. This tin was then dipped and completely immersed into a larger container filled with water for 20 s. After immersion, the water was allowed to drain off.

The weights of dehulled maize (grits) produced from the 30 kg of maize and bran produced post-dehulling and winnowing were recorded to calculate mass losses. Iron (Fe) and zinc (Zn) concentrations were determined using inductively coupled plasma optical emission spectroscopy (ICP-OES), while protein concentration was measured using a near-infrared spectrophotometric method (NIRs), both as described by Palacios-Rojas [14]. The results obtained for raw maize grain and flour was then used to calculate the percentage of nutrient concentration losses and apparent retention (AR) by comparing the concentration of the nutrient in the maize flour to that of the maize grain. True retention (TR) percentage was also reported as supplementary material, and it was calculated by multiplying the apparent retention (AR) by the proportion of dehulled grits obtained from the grain. The mass and nutriet losses were calculated on a dry weight basis. Maize hardness characterization was not conducted due to logistical challenges.

### Focus group discussions on maize flour consumption patterns

Six focus group discussions (FGDs) were conducted in rural areas of Lilongwe District, Malawi, to explore household practices, preferences, and challenges related to maize flour processing and consumption. Each FGD included 10 participants, with separate groups for men and women to ensure equitable participation and encourage open dialogue. Participant eligibility criteria included household heads or spouses of household heads and belonging to households that process their own maize flour.

### Data analysis

Shapiro–Wilk test and Bartlett’s test were used to test normality and equal variance assumptions. All assumptions were satisfied and therefore, a two-way ANOVA was used to examine the respective effects of variety and dehuller design on mass and nutrient losses, among the experiment samples, at a significance level of 5%. Post-hoc mean separations were conducted using Tukey’s honestly significant difference test where ANOVA model revealed significant differences. Narratives collected from the FGDs were examined using Thematic Analysis (TA) to identify key patterns and themes in maize flour consumption. All statistical analyses were conducted using XLSTAT (ver 2023; Addinsoft, New York, NY).

## Results

### Mass and nutrient losses during dehulling

#### Mass losses across maize varieties and dehullers

**Fig. 1:**
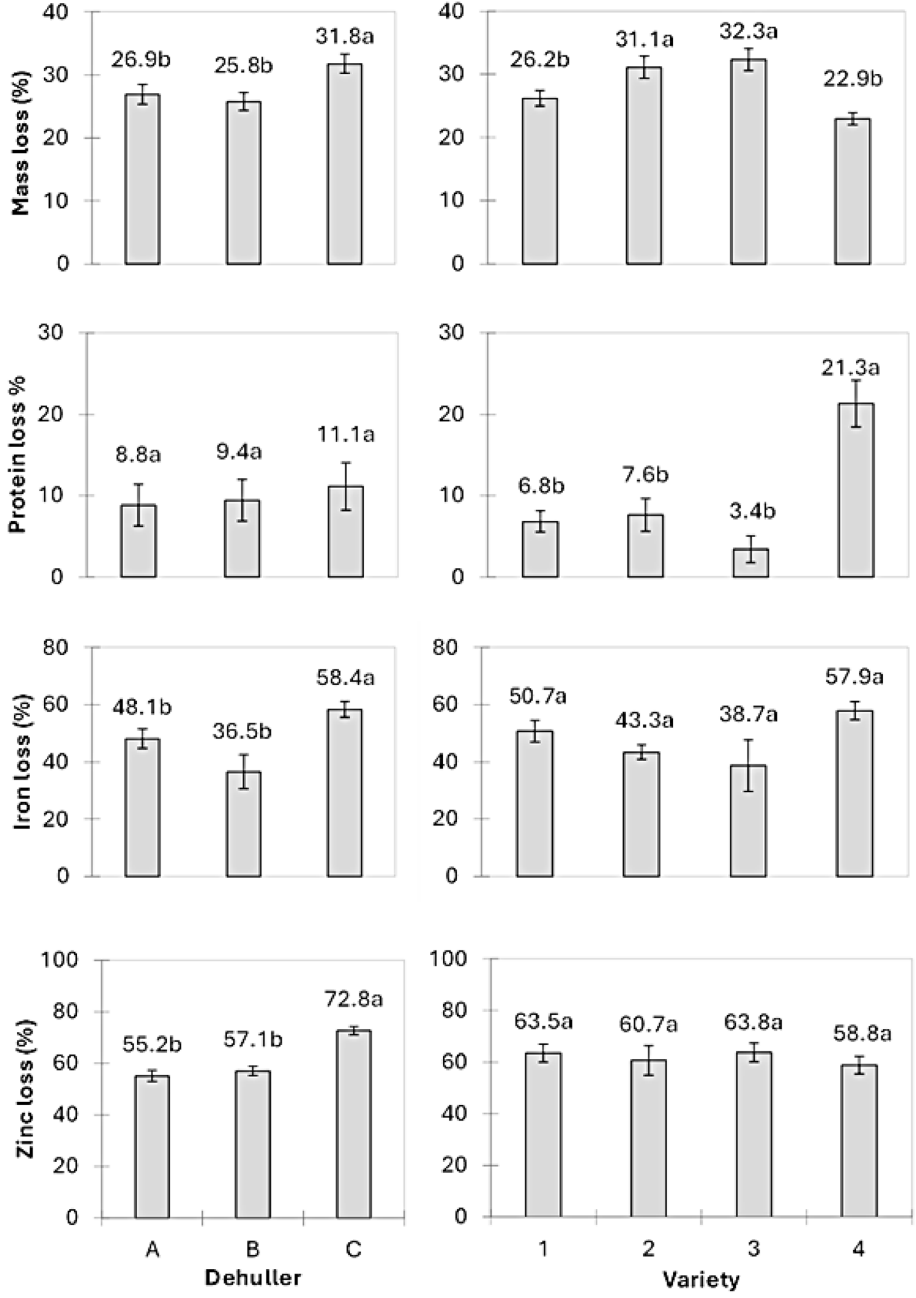
Effect of maize variety on mass loss, and protein, iron, and zinc concentration losses (%). (Varieties: 1 = Unspecified local variety (flint); 2 = Kanyani (semi-flint); 3 = Njobvu (semi-flint); 4 = Pro Vit A (flint). Error bars denote SEM

The mass losses observed among different varieties ranged from 22.9% to 32.3%, while for the different dehuller designs was 25.8–31.8% with a grand mean loss of 28.1 ± 5.7%. Analysis of the experimental data showed that both the variety of maize and dehuller design had significant effects on mass loss (*p* < 0.05). Two flint maize varieties, the local variety and Pro Vit A, along with two dehuller designs (A and B), exhibited significantly lower mass losses (Fig. 1).

#### Nutrient losses during dehulling

Initial protein, iron, and zinc levels in whole maize grains were 88 ± 2 mgg^−1^, 20.5 ± 0.4 µgg^−1^, and 18.2 ± 0.4 µgg^−1^. Dehulling resulted in the following reductions: 9.8 ± 1.9% for protein, 47.7 ± 3.6% for iron, and 61.7 ± 2.0% for zinc. Notably, the variety of maize had no significant impact on zinc loss, while the dehuller design did not significantly influence protein loss (Fig. 1).

### Maize flour consumption patterns in rural Malawi

#### Preferences for refined and whole grain flour

Members of all six FGDs reported that the majority prefer refined flour due to its long shelf life, versatility in culinary applications, and alignment with cultural dietary practices. One participant stated, “Refined flour has a longer shelf life, it stores well for a longer period and you can process for stock for the whole year.” Another added, “*Nsima* made using refined flour can easily be consumed with any other relish.”

However, participants also acknowledged the merits of whole grain flour, particularly its nutritional benefits, including higher energy content and its ability to provide satiety. One participant explained, “*Nsima* made from whole grain flour gives more energy; you can work efficiently in the field after eating it.” While refined flour is often preferred for its convenience and culinary versatility, whole grain flour stands out for its health benefits and superior nutrient content, making it especially valuable in labour-intensive settings, particularly during periods of maize scarcity.

#### Changes in flour usage

Members of all six FGDs reported that the majority switch between the flours depending on maize availability. They indicated that refined flour is commonly consumed after harvest when maize is abundant, whereas whole grain flour is used during periods of scarcity to maximize on quantity. One participant remarked, “After harvesting, when we have plenty of maize, we consume refined flour. But when maize becomes scarce, we switch to whole grain flour to stretch our reserves.”

#### Perception of whole grain flour consumption

All the six groups noted that individuals consuming whole grain flour year-round are often perceived as poor or lacking sufficient food reserves. One participant remarked, ‘We ridicule those who eat whole grain flour year-round, as it shows they are poor.’ Another added, ‘Refined flour indicates that someone has harvested a lot, whereas those eating whole grain flour are poor or lazy to process the flour.’

#### Bran utilization

All the six groups reported leaving bran at the commercial dehuller when its utility at home was limited, citing the inconvenience of carrying it. One noted, “Carrying bran back is too much work when we have no immediate use for it.” While some use bran for cooking, feeding livestock, making alcohol, or selling, its perceived value often determines whether it will be kept or left behind.

#### Awareness of losses during dehulling

Members of all six FGDs reported being aware of nutrient and volume losses during the maize dehulling process, particularly with refined flour. They noted significant reductions in flour yield and the loss of essential nutrients such as minerals and fats. A participant explained, “When you dehull maize for refined flour, you remove the bran, which reduces the volume. For example, if you take a full bag of maize to the mill, you may only return with half a bag of flour.”

#### Impact of nutritional and volume losses

Five out of the six FGDs reported that these losses contribute to household food shortages and malnutrition. They acknowledged that while bran can be repurposed, its removal deprives their diets of essential nutrients. One participant reflected, “Even though we use the bran for feeding livestock or making fertilizer, we deny our bodies the essential nutrients it contains.”

## Discussion

While the extensive mass losses due to dehulling observed in the current study might be comparable to previously published data [4], this is a significant concern in a country grappling with food insecurity. Replacing refined maize flour (*woyera*) with whole grain flour (*mgaiwa*), which avoids the 28.1% milling losses associated with dehulling, could extend household maize availability from the typical 6–7 months to approximately 9 months. Although bran from the dehulling is often used for livestock feed, many food insecure households, particularly the less privileged, either do not keep livestock or rely on free-range systems [15, 16]. Despite these losses, the culturally ingrained practice of dehulling persists, exacerbating food insecurity.

The high reduction in zinc and iron concentration due to dehulling suggests that the components with high zinc and iron concentrations (aleurone and germ) were largely removed. Considering this nutritional loss, transitioning to whole maize flour can also contribute to alleviate the prevalence of zinc deficiency problem, which currently affects 60–66% of the Malawi population across all demographic groups [17].

The 72.1% zinc loss reported during maize dehulling in the current study is concerning, given the significance of *nsima*, a maize flour-based staple food in Malawi, as a potential zinc source. Whole meal *nsima*, made from maize with an average zinc concentration of 18.2 µg/g and consumed at 383 g per capita daily (dry matter basis) [18], could provide 87% of the recommended daily zinc intake for adult females (8 mg) [19]. Similarly, whole meal *nsima* can supply 43.6% of the recommended daily iron intake for women (18 mg). However, dehulling reduces these contributions to only 33.4% for zinc and 22.8% for iron, creating significant nutritional deficits. These deficiencies are unlikely to be addressed due to the monotonous Malawian diet and limited access to alternative zinc- and iron-rich foods [20]. While these calculations may be conservative, considering that *nsima* contains phytates, which can inhibit iron absorption and affect bioavailability, these calculations nonetheless serve as a red flag on the potential losses associated with maize dehulling. Moreover, this loss not only contributes to zinc and iron deficiency but also exacerbates the protein deficiencies already prevalent in Malawi [12, 13]. Additionally, households incur additional expenses during the dehulling process, compounding the burden on a population where poverty is already widespread. However, FGDs revealed that despite awareness of these mass and nutritional losses, convenience, culinary versatility, and long shelf-life often dictate preferences for refined flour, reflecting broader structural and cultural challenges.

Although transitioning to consumption of whole instead of dehulled maize may initially pose challenges, global examples, like the Irish adoption of potatoes [21] and the introduction of maize in Africa by colonialists [22], illustrate that communities can modify dietary preferences through effective promotion despite potential cultural resistance. Emphasizing health benefits, such as reduced diabetes risk and increased dietary fibre from whole maize meal [23], or semi refined maize meal is important. While phytochemicals and antioxidants in whole grains receive less attention than those in fruits and vegetables, they are linked to decreased risks of chronic diseases, including cardiovascular disease, type 2 diabetes, cancers, and overall mortality [24]. Though it is widely acknowledged that dehulling reduces mycotoxins [25–27], reassuring consumers about readily available alternative mycotoxin prevention methods, such as sorting and pre and postharvest measures [28, 29], would alleviate concerns regarding to mycotoxin risk associated with whole grains and facilitate the transition to consumption of whole maize. Notably, Malawi’s boarding secondary schools primarily already serve *nsima* made from whole maize meal, accompanied by beans and vegetables (Author observations and experience). This existing practice offers a scalable model to normalize whole maize meal consumption. Economically disadvantaged households often blend bran with whole maize meal to produce *madeya* flour for *nsima* preparation, with the intention of augmenting its volume [30]. Moreover, the FGDs highlighted seasonal adaptations, with whole grain flour often consumed during periods of scarcity, indicating an opportunity to position it as a year-round, cost-effective, and nutritionally superior option.

While promoting the adoption of whole maize meals is important, the significant variability in mass loss observed in the current experiment highlights the urgent need for promoting more efficient milling practices. Despite significant variations in mass and nutrient losses among the three uniquely designed dehullers, all exhibited high levels of inefficiency. Additionally, their unbranded nature poses challenges for regulation and hinders efforts to promote standardized and reliable models. Introducing, branded efficient milling technologies as an intermediate strategy is equally crucial to address both mass and nutrient losses through precision engineering [31]. Furthermore, the current results underscore the importance of prioritizing micronutrient fortification of refined maize-based end-products to complement genetic maize biofortification, given the significant nutrient losses that may occur during the dehulling process. Additionally, promoting the consumption of whole grain maize, particularly high-zinc varieties, is encouraged to maximize nutrient intake especially in regions of the country where zinc-deficient soils are prevalent [32].

## Conclusions

Our investigation provided significant insights into our hypotheses. The results confirmed that maize variety and dehuller design significantly influence nutrient losses, particularly for protein, zinc, and iron. The FGDs supported the hypothesis that consumer preferences for refined versus whole maize flour are shaped by cultural norms, poverty perceptions, and seasonal maize availability. These findings underscore the multifaceted challenge of addressing mass and nutrient losses during dehulling.

While breeding maize varieties with higher dehulling yields and developing dehullers that minimize nutrient losses are promising solutions, their practical feasibility is constrained by resource limitations and varying consumer preferences. A more immediate and scalable strategy lies in promoting whole maize meal consumption as a culturally acceptable and nutritionally superior alternative. This approach requires addressing deeply ingrained cultural stigma and poverty associations linked to whole grain flour.

Tailored campaigns and community engagement initiatives are essential to shift these narratives, focusing on the health benefits, economic advantages, and sustainability of whole maize meal. For communities resistant to whole grain adoption, improving dehulling and degerming technologies to retain more nutrients while maintaining desirable flour characteristics is a critical complementary strategy.

## Supplementary Information

Below is the link to the electronic supplementary material.

Supplementary file 1 (DOCX 348 KB)

## Data Availability

The raw data supporting the conclusions of this article will be made available by the authors on request.
