# Supplementary material for: Whole maize flour could enhance food and nutrition security in Malawi
Source: Discov Food. 2025 Feb 17;5(1):40. doi: 10.1007/s44187-025-00311-y (PMC11832564; doi:10.1007/s44187-025-00311-y)
Supplement: Supplementary file 1 — (DOCX 348 KB) [file 44187_2025_311_MOESM1_ESM.docx]

**SUPPLEMENTARY MATERIAL**

| **Dehuller** | **Photo** |
| --- | --- |
| A | 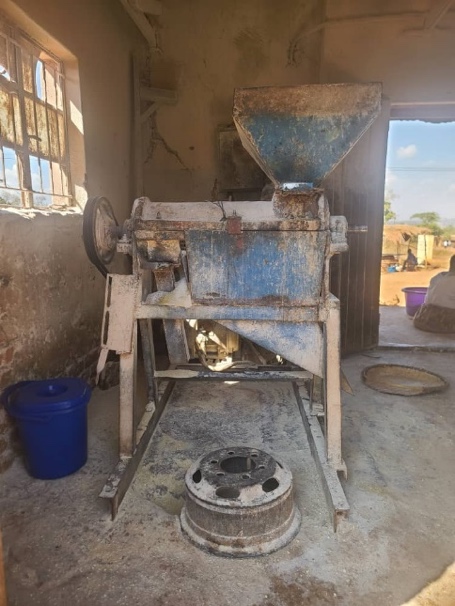 |
| B | 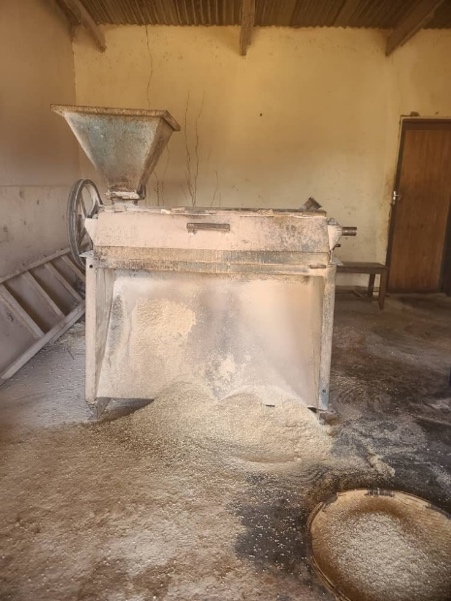 |
| C | 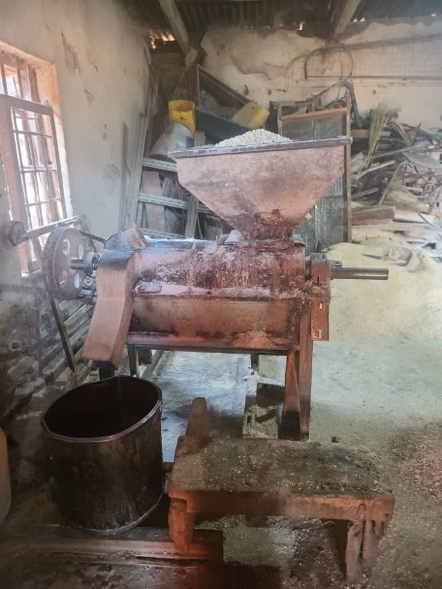 |

Suppementary Figure 1: Unbranded locally manufactured dehullers used in the study
